# Using a smartwatch and smartphone to assess early Parkinson’s disease in the WATCH-PD study

**DOI:** 10.1038/s41531-023-00497-x

**Published:** 2023-04-17

**Authors:** Jamie L. Adams, Tairmae Kangarloo, Brian Tracey, Patricio O’Donnell, Dmitri Volfson, Robert D. Latzman, Neta Zach, Robert Alexander, Peter Bergethon, Joshua Cosman, David Anderson, Allen Best, Joan Severson, Melissa A. Kostrzebski, Peggy Auinger, Peter Wilmot, Yvonne Pohlson, Emma Waddell, Stella Jensen-Roberts, Yishu Gong, Krishna Praneeth Kilambi, Teresa Ruiz Herrero, E. Ray Dorsey, the Parkinson Study Group Watch-PD Study Investigators and Collaborators, Jamie L. Adams, Christopher Tarolli, Emma Waddell, Stella Jensen-Roberts, Julia Soto, Penelope Hogarth, Mastura Wahedi, Katrina Wakeman, Alberto J. Espay, Julia Brown, Christina Wurzelbacher, Steven A. Gunzler, Elisar Khawam, Camilla Kilbane, Meredith Spindler, Megan Engeland, Arjun Tarakad, Matthew J. Barrett, Leslie J. Cloud, Virginia Norris, Zoltan Mari, Kara J. Wyant, Kelvin Chou, Angela Stovall, Cynthia Poon, Tanya Simuni, Kyle Tingling, Nijee Luthra, Caroline Tanner, Eda Yilmaz, Danilo Romero, Karen Thomas, Leslie Matson, Lisa Richardson, Michelle Fullard, Jeanne Feuerstein, Erika Shelton, David Shprecher, Michael Callan, Andrew Feigin, Caitlin Romano, Martina Romain, Michelle Shum, Erica Botting, Leigh Harrell, Claudia Rocha, Ritesh Ramdhani, Joshua Gardner, Ginger Parker, Victoria Ross, Steve Stephen, Katherine Fisher, Jeremy Edgerton, Jesse Cedarbaum, Robert Rubens, Jaya Padmanabhan, Diane Stephenson, Brian Severson, Michael Merickel, Daniel Jackson Amato, Thomas Carroll

**Affiliations:** 1 Center for Health + Technology, University of Rochester Medical Center, Rochester, NY USA; 2 Department of Neurology, University of Rochester Medical Center, Rochester, NY USA; 3 Takeda Pharmaceuticals, Cambridge, MA USA; 4 Sage Therapeutics, Seattle, WA USA; 5 Banner Health, Phoenix, AZ USA; 6 Invariant Research Limited, Dover, MA USA; 7 AbbVie Pharmaceuticals, North Chicago, IL USA; 8 Clinical Ink, Horsham, PA USA; 9 Department of Radiology, Massachusetts General Hospital, Harvard Medical School, Boston, MA USA; 10 Athinoula A. Martinos Center for Biomedical Imaging, Massachusetts General Hospital, Harvard Medical School, Massachusetts Institute of Technology, Boston, MA USA; 11 Bill and Melinda Gates Foundation, Seattle, WA USA; 12 University of Rochester, Rochester, NY USA; 13 Oregon Health and Science University, Portland, OR USA; 14 University of Cincinnati, Cincinnati, OH USA; 15 University Hospitals Cleveland Medical Center, Cleveland, OH USA; 16 University of Pennsylvania, Philadelphia, PA USA; 17 Baylor College of Medicine, Houston, TX USA; 18 Virginia Commonwealth University, Richmond, VA USA; 19 Cleveland Clinic Lou Ruvo Center for Brain Health, Las Vegas, NV USA; 20 University of Michigan, Ann Arbor, MI USA; 21 Northwestern University, Evanston, IL USA; 22 University of California, San Francisco, CA USA; 23 Sentara Neurology Specialists, Virginia Beach, VA USA; 24 University of Colorado Denver, Denver, CO USA; 25 Banner Research Institute, Phoenix, AZ USA; 26 New York University, New York, NY USA; 27 University of South Florida, Tampa, FL USA; 28 Northwell, New Hyde Park, NY USA; 29 Center for Health + Technology, Rochester, NY USA; 30 Biogen, Cambridge, MA USA; 31 Critical Path Institute, Tucson, AZ USA; 32 Digital Artefacts/Clinical Ink, Iowa City, IA USA

**Keywords:** Parkinson's disease, Parkinson's disease

## Abstract

Digital health technologies can provide continuous monitoring and objective, real-world measures of Parkinson’s disease (PD), but have primarily been evaluated in small, single-site studies. In this 12-month, multicenter observational study, we evaluated whether a smartwatch and smartphone application could measure features of early PD. 82 individuals with early, untreated PD and 50 age-matched controls wore research-grade sensors, a smartwatch, and a smartphone while performing standardized assessments in the clinic. At home, participants wore the smartwatch for seven days after each clinic visit and completed motor, speech and cognitive tasks on the smartphone every other week. Features derived from the devices, particularly arm swing, the proportion of time with tremor, and finger tapping, differed significantly between individuals with early PD and age-matched controls and had variable correlation with traditional assessments. Longitudinal assessments will inform the value of these digital measures for use in future clinical trials.

## Introduction

Parkinson’s disease (PD) is the world’s fastest-growing neurological disorder^1^. Despite increasing prevalence and substantial investment from private and public funders, however, therapeutic breakthroughs have been scant this century, especially for early disease^2^. While rating scales have improved, they still provide subjective, episodic, and largely insensitive assessments contributing to large, lengthy, expensive trials that are prone to failure^3,4^. Moreover, scales like the Movement Disorders Society Unified Parkinson’s Disease Rating Scale (MDS-UPDRS) Part III have high inter-observer variability and have faced challenges in detecting disease progression in neuroprotective trials^5^. In addition, these scales may not accurately assess the patient experience. Better measures could lead to more efficient, patient-centric, and timely evaluation of therapies.

Digital tools can provide objective, sensitive, real-world measures of PD^4,6–8^. A smartphone research application, previously used in phase 1 and 2 clinical trials, differentiated individuals with early PD from age-matched controls through finger tapping and detected tremor not apparent to investigators^9,10^. Similarly, a smartwatch measured tremor and detected motor fluctuations and dyskinesias^11^.

Despite this promising pilot data, few studies have assessed multiple digital devices in a multicenter study that replicates a clinical trial setting in individuals with early, untreated PD. We sought to evaluate the ability of research-grade wearable sensors, a smartwatch and a smartphone to assess key features of PD. We used a platform specifically designed to incorporate several assessments that map directly onto the MDS-UPDRS, providing an objective digital analog to subjective clinical measurements. We aimed to determine the specific disease features these digital tools can detect, whether the measures differed between individuals with early PD and age-matched controls, and how well the digital measures correlated with traditional ones. Here, we report the results of the baseline analyses of a 12-month longitudinal study, focused on the smartphone application and smartwatch results from the first in clinic visit and at-home passive monitoring period following that visit.

## Results

### Study participants

Eighty-two individuals with early, untreated PD and 50 age-matched controls completed informed consent to participate in this WIRB-Copernicus Group (WCG)^TM^ Institutional Review Board approved study at 17 research sites between June 2019 and December 2020 (Supplemental Fig. 1). Participants with PD were more likely to be men and were similar to those in the PPMI study (Table 1)^3,12–15^.

**Table 1:** Baseline characteristics of research participants in this study and de novo Parkinson’s disease participants in the Parkinson’s Progression Markers Initiative.

	Characteristic	PD cohort (*n* = 82)	Control cohort (*n* = 50)	*p* value	PPMI de novo cohort (*n* = 423)
**Demographic characteristics**	Age, y	63.3 (9.4)	60.2 (9.9)	0.07	61.7 (9.7)
	Male, *n* (%)	46 (56)	18 (36)	0.03	277 (65)
	Race, *n* (%)			0.81	
	White	78 (95)	48 (96)		391 (92)
	Black or African American	0 (0.0)	0 (0)		6 (1)
	Asian	3 (4)	1 (2)		8 (2)
	Not specified	1 (1)	1 (2)		18 (4)
	Hispanic or Latino, *n* (%)	3 (4)	1 (2)	0.99	9 (2)
	Education >12 Years, *n* (%)	78 (95)	48 (96)	0.99	347 (82)
**Clinical characteristics**	Right or mixed handedness, *n* (%)	74 (90)	47 (94)	0.53	385 (91)
	Parkinson’s disease duration, months	10.0 (7.3)	N/A	N/A	6.7 (6.5)
	Hoehn & Yahr, *n* (%)			<0.001	
	Stage 0	0 (0)	49 (100)		0 (0)
	Stage 1	19 (23)	0 (0)		185 (44)
	Stage 2	62 (76)	0 (0)		236 (56)
	Stage 3-5	1 (1)	0 (0)		2 (0.5)
	MDS-UPDRS				
	Total Score	35.2 (12.4)	5.9 (5.3)	<0.001	32.4 (13.1)
	Part I	5.5 (3.6)	2.8 (2.6)	<0.001	5.6 (4.1)
	Part II	5.6 (3.8)	0.4 (1.0)	<0.001	5.9 (4.2)
	Part III	24.1 (10.2)	2.7 (3.5)	<0.001	20.9 (8.9)
	Montreal Cognitive Assessment	27.6 (1.4)	28.1 (1.5)	0.04	27.1 (2.3)
	Parkinson’s Disease Quality of Life Questionnaire	7.7 (6.7)	N/A	N/A	N/A
	Geriatric Depression Scale (Short Version)	1.6 (1.9)	1.0 (1.2)	0.05	2.3 (2.4)
	REM Sleep Behavior Disorder Questionnaire	4.4 (3.1)	2.7 (2.0)	<0.001	4.1 (2.7)
	Epworth Sleepiness Scale	4.9 (3.2)	4.6 (3.7)	0.66	5.8 (3.5)
	Scale for Outcomes in Parkinson’s Disease for Autonomic Symptoms	9.1 (5.1)	5.3 (4.2)	<0.001	9.5 (6.2)

*PD* Parkinson’s disease, *PPMI* Parkinson’s Progression Markers Initiative, *N/A* Not available, *MDS-UPDRS* Movement Disorder Society-Unified Parkinson’s Disease Rating Scale.

Results are mean (standard deviation) for continuous measures and *n* (%) for categorical measures.

One control cohort participant is missing the Hoehn & Yahr and MDS-UPDRS scores and one additional is missing the MDS-UPDRS part III and total scores.

### Gait

**Fig. 1:**
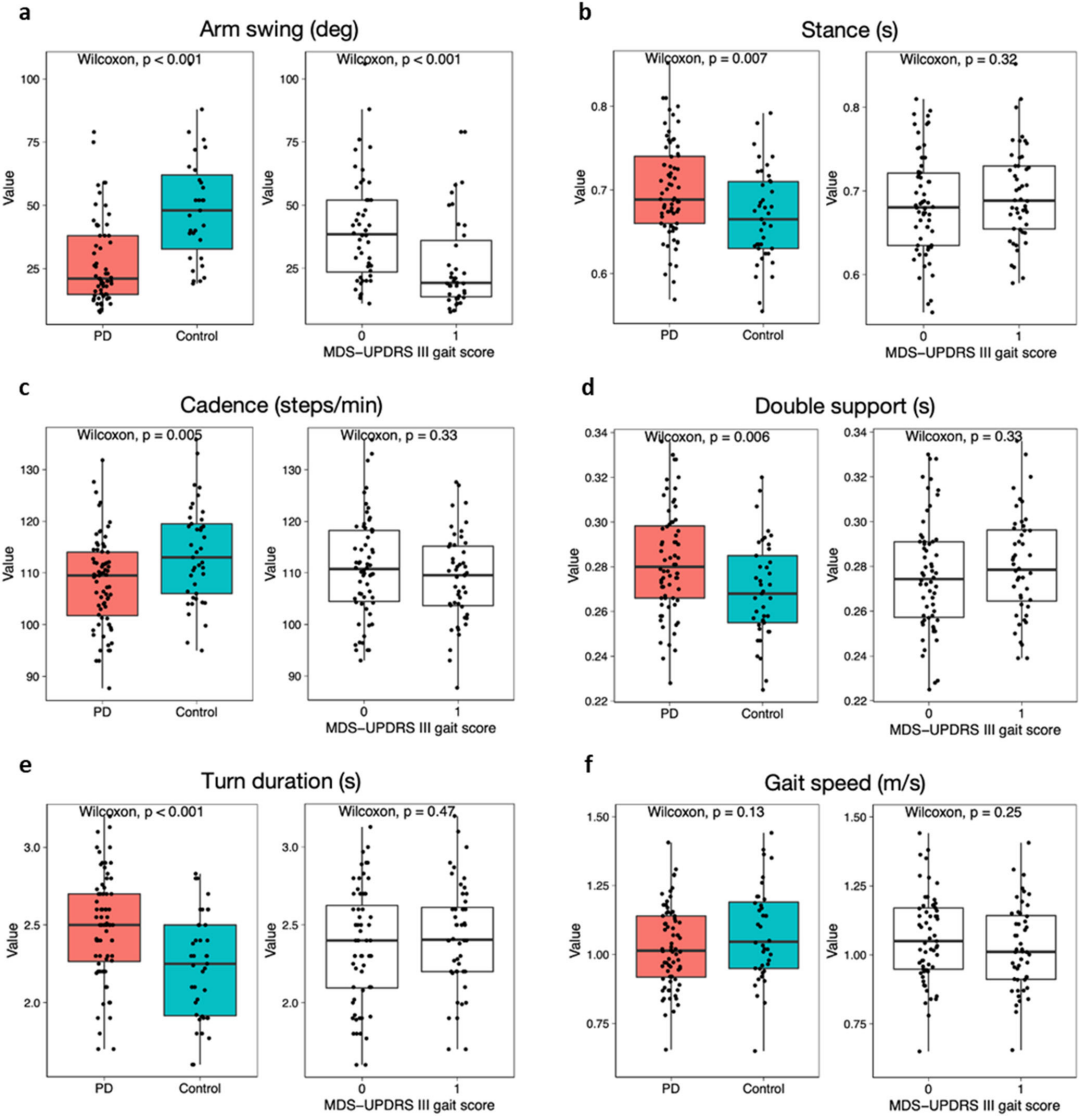
Comparisons of gait features derived from a smartphone and smartwatch. Box plots for (panel **a**) Arm swing (deg), (panel **b**) Stance (s), (panel **c**) Cadence (steps/min), (panel **d**) Double support (s), (panel **e**) Turn duration (s), and (panel **f**) Gait speed (m/s) between those with and without Parkinson’s disease and by MDS-UPDRS part III gait item 3.10. MDS-UPDRS Movement Disorder Society-Unified Parkinson’s Disease Rating Scale.

Smartphone data were available for gait analysis for 72 participants with PD and 41 controls; smartwatch data were available for at least 59 PD participants and at least 31 controls. Based on data from the baseline clinic visit, one gait parameter from the smartwatch and five from the phone best differentiated those with PD from controls. The magnitude of arm swing (Fig. 1a), as measured by the smartwatch, was smaller in PD than controls (27.8 [17.0] degrees vs 49.2 [21.8] degrees; *P* < 0.001). The smartphone detected increased stance time, slower gait cadence, increased total double support time, and increased turn duration among PD participants (Fig. 1b–e). Gait speed (Fig. 1f) did not differ between the two groups (1.03 [0.15] m/s vs 1.05 [0.24] m/s; *P* = 0.13). The smartphone also detected increased initial double support time in individuals with PD (data not shown). Only the magnitude of arm swing (*P* < 0.001) and stride length variability (*P* = 0.01) showed separation between controls and PD when grouped based on the MDS-UPDRS part III gait score (item 3.10). The gait differences observed between PD participants and controls were generally smaller among women than men (data not shown). Gait parameters derived from the smartwatch and smartphone showed moderate to very strong correlations (0.36 < r < 0.79) with comparable metrics from the research-grade wearable sensors.

### Psychomotor function

**Fig. 2:**
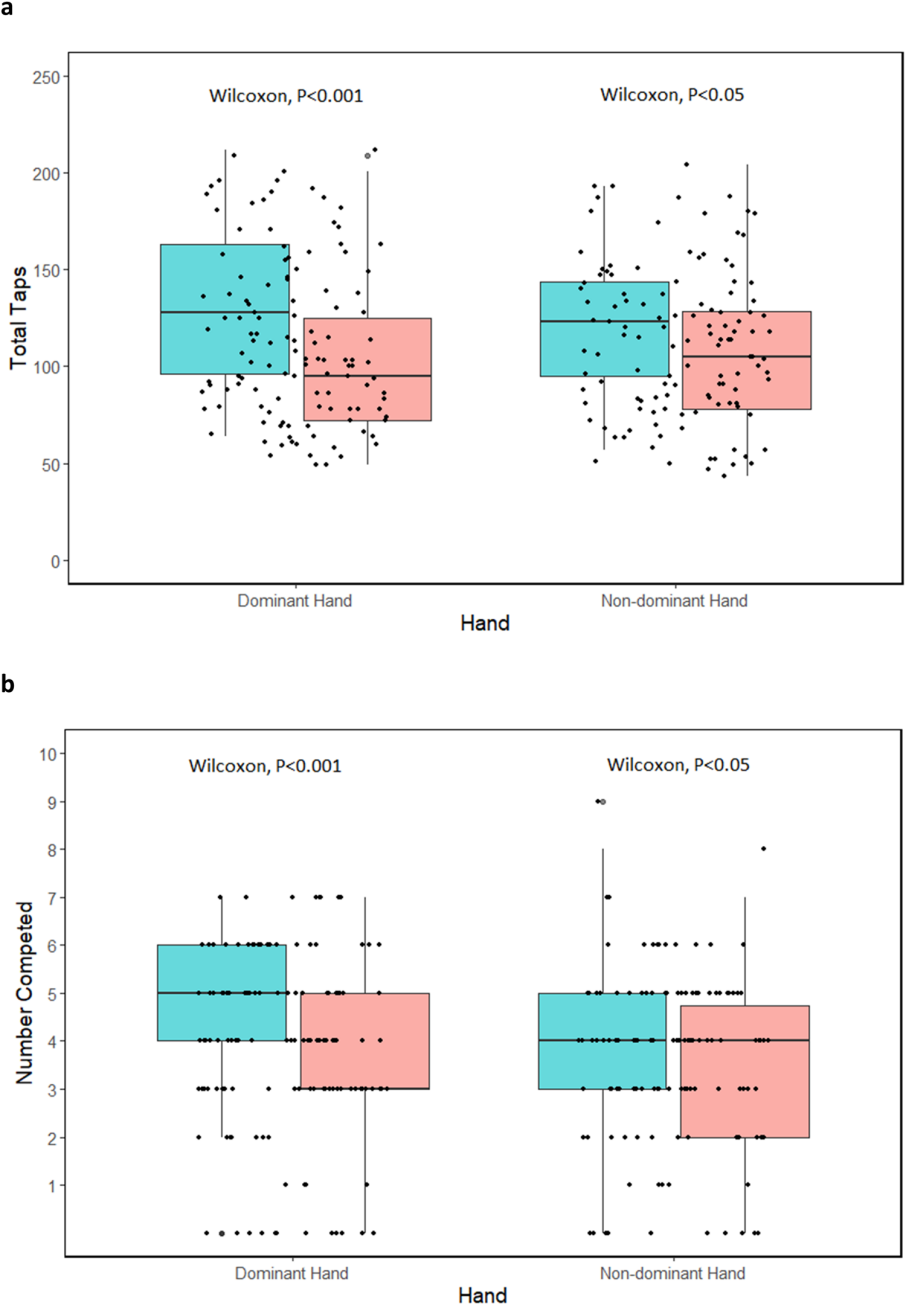
Comparisons of psychomotor function derived from a smartphone. Box plots for (panel **a**) number of total finger taps and (panel **b**) number of objects successfully manipulated on a smartphone between those with and without Parkinson’s disease by dominant and non-dominant hand.

Smartphone data were available for analysis for 78 participants with PD and 45 controls for finger-tapping, and 82 participants with PD and 49 controls for the fine motor task. Finger tapping in the dominant and nondominant hand was slower in PD participants than controls (104.5 [40.5] taps per 30 s vs 130.2 [40.9] taps per 30 s; *P* < 0.001; and 106.4 [39.9] taps vs 122.2 (34.6) taps; *P* = 0.02, respectively) (Fig. 2a). Significant differences in total taps were also seen in PD participants when looking at most affected versus least affected side (98.6 [37.9] versus 112.3 [42.2]; *P* < 0.05). The inter-tap interval, or time between each tap, was longer in individuals with PD than controls in their dominant (169.9 [68.2] ms vs 137.3 (38.4) ms; *P* = .008) and nondominant hand (173.0 [67.7] ms vs 141.9 (32.1) ms; *P* = 0.02). The inter-tap interval coefficient of variation was also greater in PD in the dominant (50.4% [0.2] vs 32.1% [0.1]; *P* < 0.001) and non-dominant hand (50.8% [0.2] vs 36.2% [0.1]; *P* < 0.001). Slower tapping speeds very weakly correlated with higher scores on the MDS-UPDRS finger tapping for the right (r = −0.19) and left (r = −0.10) hands. The number complete in the fine-motor test was significantly less in individuals with PD than controls in their dominant (3.4 [1.7] vs 4.6 [1.9]; *P* < 0.001) and non-dominant hand (3.4 [1.7] vs 4.0 [1.9]; *P* < 0.05) (Fig. 2b). There were no significant differences in PD participants in total complete when examining most affected versus least affected side.

### Tremor

**Fig. 3:**
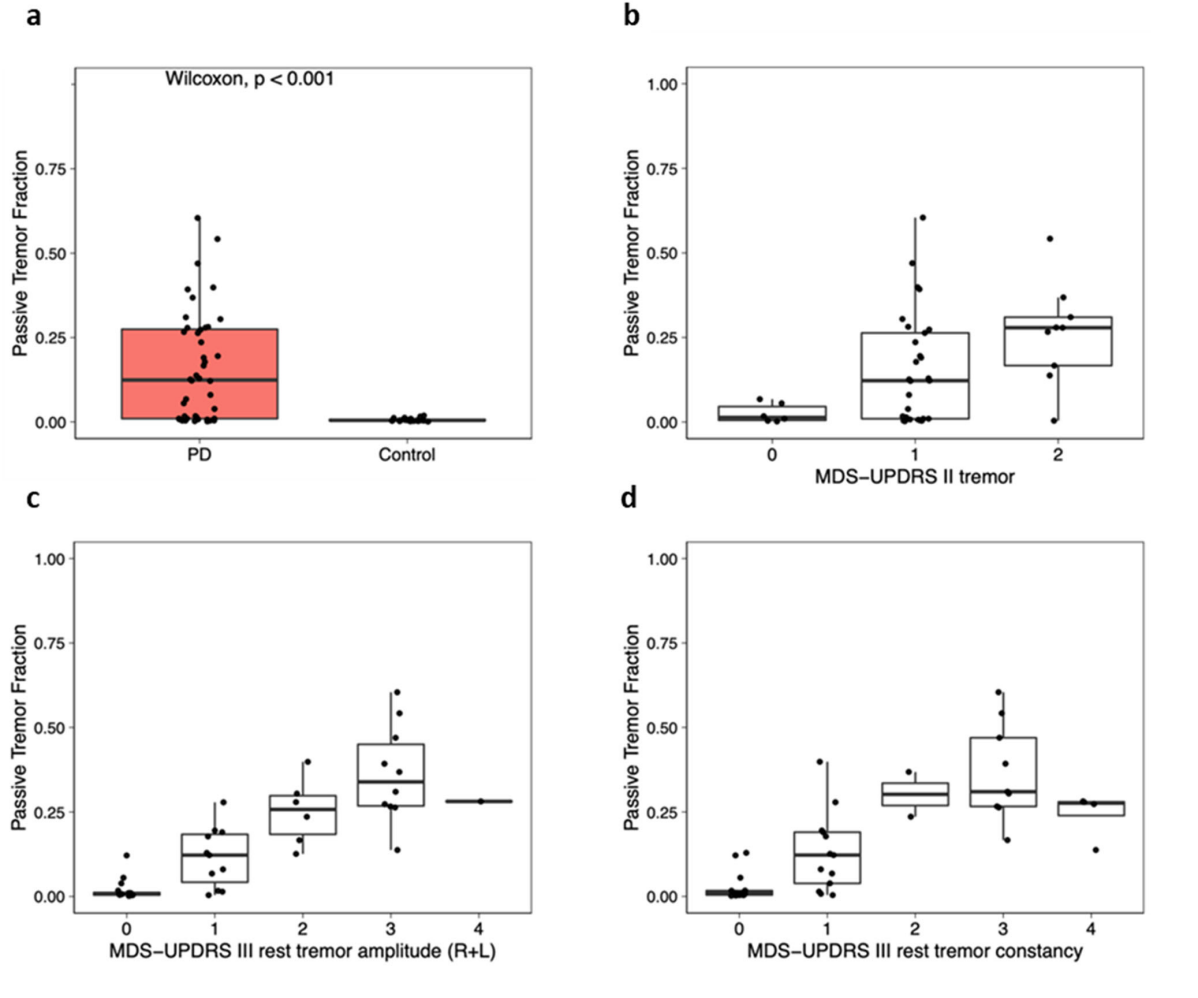
Comparisons of tremor scores derived from continuous passive tremor data from a smartwatch. Box plots for passive tremor fraction between (panel **a**) those with and without Parkinson’s disease and by (panel **b**) MDS-UPDRS part II tremor, (panel **c**) MDS-UPDRS part III rest tremor amplitude (right + left), and (panel **d**) MDS-UPDRS part III rest tremor constancy. MDS-UPDRS Movement Disorder Society-Unified Parkinson’s Disease Rating Scale.

Passive tremor classification data from the smartwatch were available for 44 participants with PD and 22 controls for the at-home monitoring period following the baseline visit. The proportion of time with tremor (“passive tremor fraction”) was significantly higher among participants with PD (15.9% (16.3)) compared to controls (0.6% (0.5); *P* < 0.001) (Fig. 3a). Among PD participants, the tremor fraction measured correlated moderately with self-reported tremor severity (MDS-UPDRS part II, item 10, r = 0.43, *p* = 0.003), very strongly with clinician-reported upper extremity rest tremor amplitude (MDS-UPDRS part III, item 17, r = 0.86, *p* < 0.001), and strongly with rest tremor constancy (MDS-UPDRS part III, item 18, r = 0.79, *p* < 0.001, Fig. 3b–d).

### Cognition

Data on smartphone cognitive tests were available for 82 participants with PD and 49 controls. PD participants performed worse on the Trail Making Test Part A (54.5 (23.8) vs 48.0 (36.0) seconds; *P* < 0.05) and had fewer correct responses (18.3 (8.2) vs 20.4 (8.9); *P* = 0.05) on the Symbol Digit Modalities Test than controls. Higher scores on the Montreal Cognitive Assessment correlated weakly with decreased time to complete Trails A (r = −0.20, *P* = 0.14) and Trails B (r = −0.38, *P* < 0.01), more correct matches on the Symbol Digital Modalities Test (r = 0.25, *P* < 0.05), and the proportion of correct answers on the Visuospatial Working Memory Test (r = 0.18, *P* = 0.16).

### Speech

**Fig. 4:**
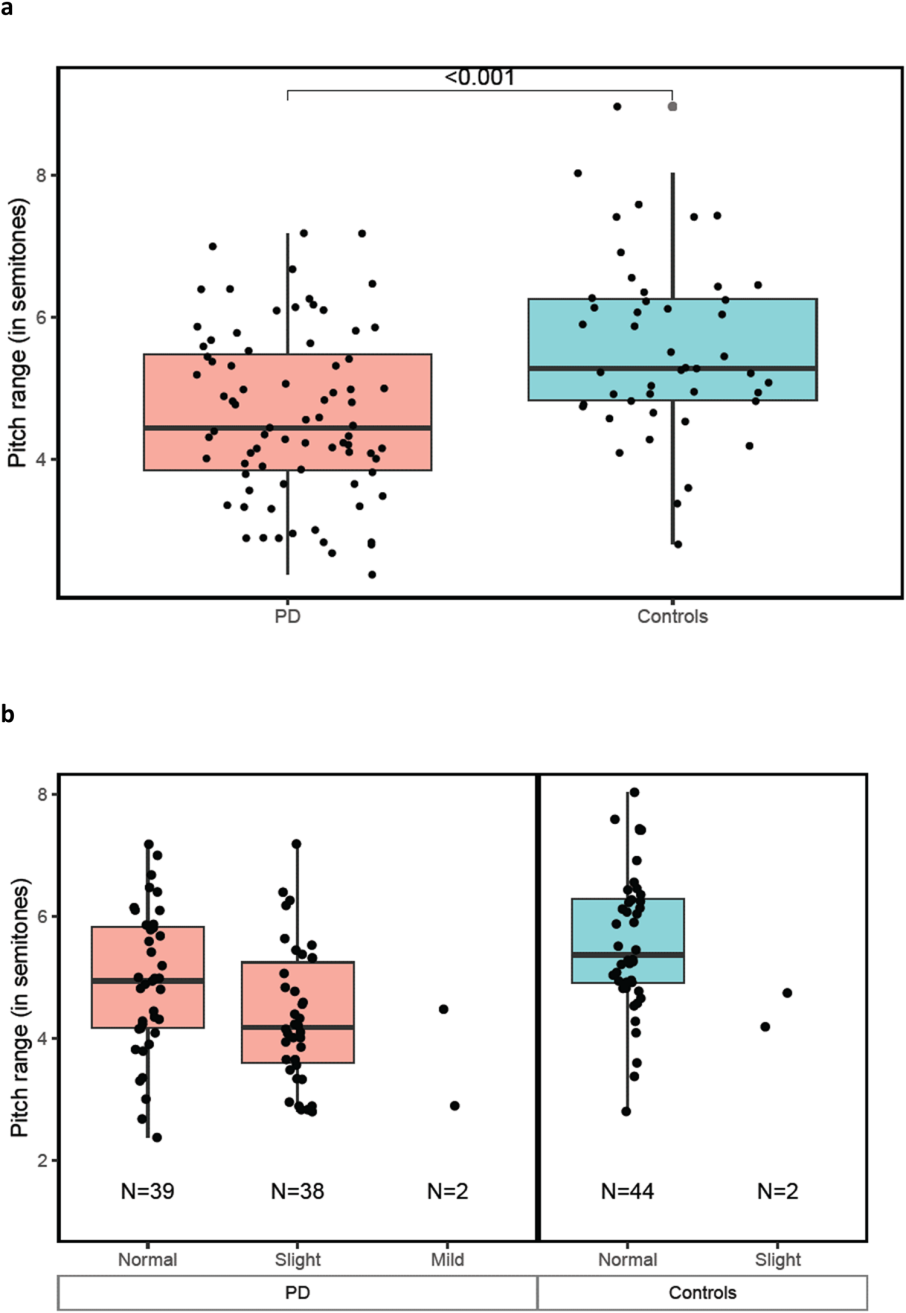
Comparisons of speech performance derived from a smartphone. Box plots for pitch range (in semitones) between (panel **a**) those with and without Parkinson’s disease and by (panel **b**) MDS-UPDRS speech item 3.1. MDS-UPDRS Movement Disorder Society-Unified Parkinson’s Disease Rating Scale.

Baseline reading task data were available from 79 PD participants and 46 controls. Phonation task data were analyzed for 53 PD participants and 41 controls. In the reading task, the average pitch range (measured in semitones) for PD participants was reduced compared to controls (4.6 [1.2] vs 5.6 [1.2]; *P* = 0.00004) (Fig. 4). This was also true for individuals with PD who were rated as having “normal” speech on the MDS-UPDRS, (4.9 [1.2] vs 5.6 [1.2]; *p* = 0.015). Speech from reading and phonation tasks differed between those with and without PD (Supplemental Table 1). The most distinctive speech features varied with sex. For women, the median pause duration in the reading task best distinguished PD from controls (AUC = 0.72). For men, this distinction was best made by pitch semitone range in the reading task (AUC = 0.79).

## Discussion

In this multicenter study, a commercially available smartwatch and a smartphone research application captured key motor and non-motor features of early, untreated PD. These measures differed from age-matched controls, had variable correlation with traditional measures, and offer the promise of objective, real-world measures of the disease for use in future studies.

Compared to other digital studies in PD, this study has several strengths. The study population, which came from a network that often conducts PD trials, consisted of individuals with early, untreated PD, a target population for disease-modifying therapies. This study focused on deriving key features from a specialized software battery of assessments installed on commercially available devices, which have several advantages^16,17^. The devices are familiar to many, user friendly, have standardized software upgrades, enable remote data capture, and can inform individuals of results^11^. Devices and data plans were provided to minimize the effects of variable access to technology or the internet based on socioeconomic status or geographic location. Perhaps reflecting these advantages, interest among sites and participants was high, and enrollment was completed in a timely manner despite the COVID-19 pandemic. Finally, using a combination of devices both in the clinic and at home, the study collected a wide range of digital measures, which longitudinally evaluate a broad spectrum of motor and non-motor symptoms.

The study results are largely consistent with previous studies using other digital devices. For example, the reduced arm swing is a common early feature of PD that has been detected by sensing cameras and may even be a prodromal feature that can be measured with wearable sensors^18,19^. Similarly, slower finger tapping, increased tremor, worse cognition, and speech changes in PD compared to controls have all been demonstrated by different devices^9,11,20–23^.

Consistent with a previous study, digital devices were more sensitive than rating scales for some measures^24^. For example, the smartphone application detected abnormalities in speech even when it was rated “normal” by investigators. The better sensitivity of digital measures may explain the variable correlation with traditional clinical measures. For instance, the “inter-tap interval coefficient of variation” is not reliably weighed on the MDS-UPDRS, which does not separate speed from dysrhythmia. This study, like others, also found differences by sex, including in speech, and shows the value of connected speech tasks^25,26^. More research is needed, including longitudinal analysis from this study, to determine which measures are most sensitive to change over time.

This study was limited by missing data, wearing of the watch on different sides, lack of familiarity with some tasks, the homogeneous study population, and questions about the meaningfulness of the measures. Digital devices provide large volumes of data, but like imaging studies, can be prone to missing data^27^. For some assessments, data from more than half of the participants were not available. The biggest loss was due to device permissions restrictions, which were inadvertently turned off in some devices, impeding the transfer of passive data from the smartwatch to the analytical database. This shortcoming limits statistical power and highlights the need for rigorous data management and monitoring throughout the study.

In WATCH-PD, the smartwatch was worn on only one wrist. The side worn by PD participants (more affected side) and controls (individual preference) differed and at times was inconsistent with actual use. Standardizing wear for all participants to one side may be simpler and provide more consistent data. Some app tasks were also new to participants. This novelty may have contributed to more modest correlations than seen in previous studies and could have benefitted from practice prior to the baseline assessment^9^.

Like many other digital studies and PD trials, participants in this study were overwhelmingly white and well-educated, and thus not representative of the general population^28–30^. Empowering participants with data and active study roles and ensuring that investigators and sites are trustworthy, accessible, and representative of the broader population can make research more inclusive and equitable^31–33^. Given that this study evaluated individuals with early PD who had mild motor impairment and excluded those with cognitive impairment, the digital tasks may not be feasible in more advanced populations.

Regulators in Europe and the U.S. have recently accepted measures of gait speed in Duchenne muscular dystrophy and moderate to vigorous physical activity in idiopathic pulmonary fibrosis as endpoints for clinical trials^34,35^. These endpoints, which could be derived from this cohort, may also be valuable in PD. Other measures not included in this study, like step counts or words spoken, may also be collected by digital technologies and are not easily measured clinically. For use as endpoints in clinical trials, digital measures should be sensitive to change. Longitudinal data from this study are forthcoming and will help determine each measure’s sensitivity to change. Most importantly, digital measures must be meaningful. To determine which measures are meaningful, the voice of the patient must be included. The U.S. Food and Drug Administration has undertaken such efforts for idiopathic pulmonary fibrosis, and we are conducting qualitative research with participants from this study to understand what digital measures are relevant to their symptoms^36^.

This multicenter study in early, untreated PD provides valuable data on multiple digital measures derived from widely available devices. We were able to detect motor and non-motor features that differed between individuals with early PD and age-matched controls. In some cases, the digital devices were more sensitive than clinician-dependent rating scales. Longitudinal analysis as well as participant input will help identify potential digital measures to evaluate much-needed therapies for this rapidly growing population.

## Methods

### Study design

WATCH-PD (Wearable Assessment in The Clinic and at Home in PD)(NCT03681015) is a 12-month, multicenter observational study that evaluated the ability of digital devices to assess disease features and progression in persons with early, untreated PD.

### Ethics

The WCG^TM^ Institutional Review Board approved the procedures used in the study, and there was full compliance with human experimentation guidelines.

### Setting

The study population was recruited from clinics, study interest registries, and social media and enrolled at 17 Parkinson Study Group research sites. All participants with PD had diagnoses confirmed clinically by a movement disorders specialist with approximately half undergoing a screening dopamine transporter imaging (DaTscan) to confirm diagnosis via imaging. Participants were evaluated in clinic and at home. In-person visits occurred at screening/baseline and then at months 1, 3, 6, 9, and 12. Due to the COVID-19 pandemic, most month-3 visits were converted to remote visits via video or phone, and participants could elect to complete additional visits remotely.

### Participants

We sought to evaluate a population similar to the Parkinson’s Progression Markers Initiative (PPMI)^37^, which is a target population of trials evaluating disease-modifying therapies. For those with PD, the principal inclusion criteria were age 30 or greater at diagnosis, disease duration less than two years, and Hoehn & Yahr stage two or less. Exclusion criteria included baseline use of dopaminergic or other PD medications and an alternative parkinsonian diagnosis. Control participants without PD or other significant neurologic diseases were age-matched to the PD cohort. All participants provided written informed consent before study participation.

### Data sources/measurement

Supplemental Table 2 outlines the study’s gait, motor function, tremor, cognitive, and speech assessments.

**Fig. 5:**
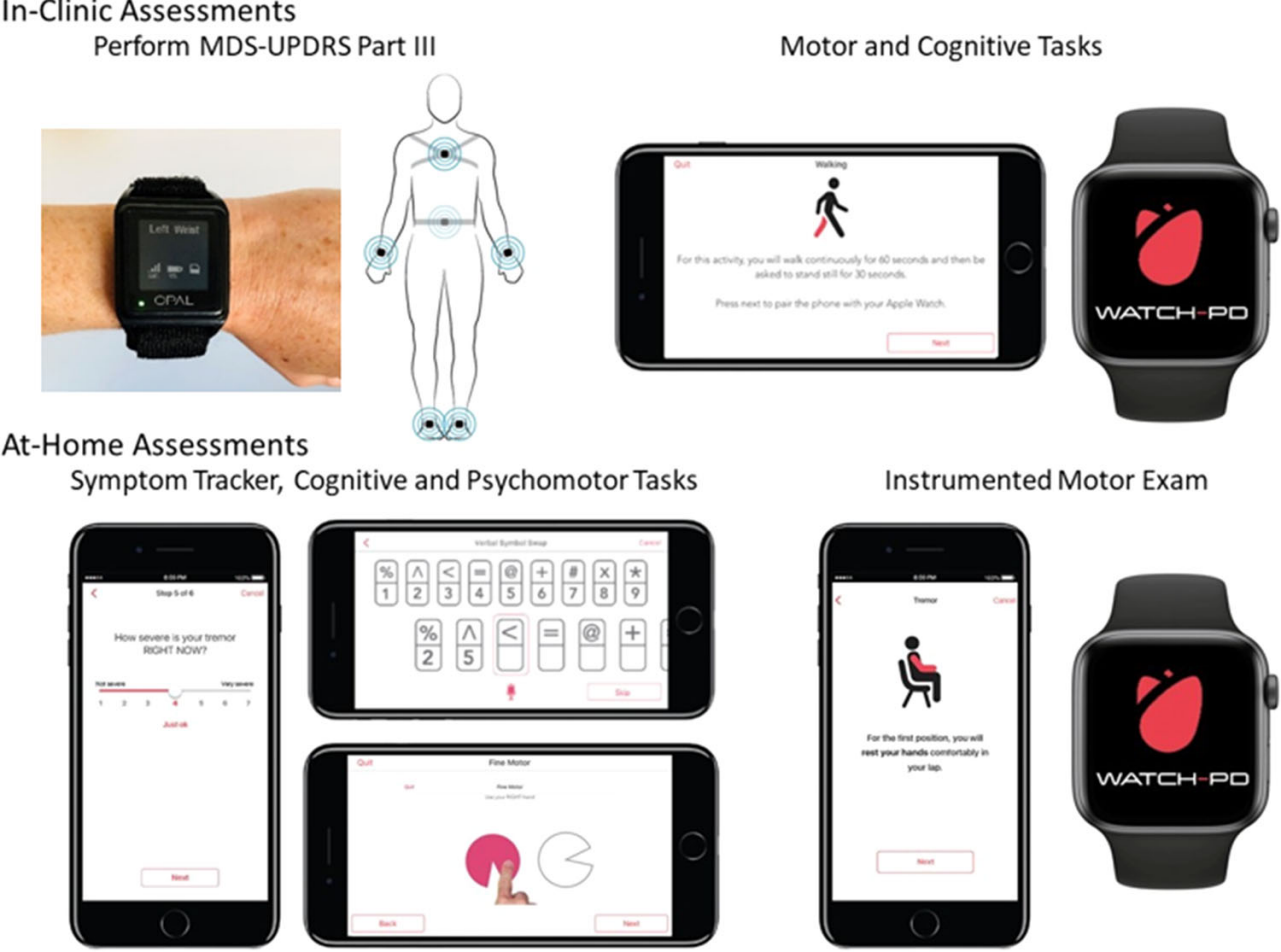
Digital devices evaluated in-clinic and at-home during the study. MDS-UPDRS Movement Disorder Society-Unified Parkinson’s Disease Rating Scale. Copyrighted images of BrainBaseline’s Movement Disorders Mobile Application were reprinted with permission by Clinical ink (Horsham, PA). Copyrighted images of APDM Wearable Technology were reprinted with permission by APDM Wearable Technologies, a Clario Company (Portland, OR).

This study used three devices (Fig. 5): research-grade wearable “Opal” sensors (APDM Wearable Technologies, a Clario Company), an Apple Watch 4 or 5, and an iPhone 10 or 11 (Apple, Inc.) running a smartphone application specifically for PD (BrainBaseline™). Raw mobility and speech signals were recorded from Apple’s native accelerometer (100 Hz sampling rate) and microphone (32 kHz sampling rate) hardware configurations.

During in-clinic visits, six wearable sensors with an accelerometer, gyroscope, and magnetometer were placed on the sternum, lower back, and on each wrist and foot. Smartphone application tasks were conducted at each clinic visit and at home every two weeks on the smartphone. The smartphone was worn in a lumbar sport pouch during gait and balance tests.

Gait features were extracted from the smartwatch and smartphone using software developed in-house. Gait bouts were identified after turns, and gait features during each bout were extracted using open-source GaitPy^38,39^. Arm swing features were calculated using rotational velocity from the smartwatch. Movement data was collected from the wearable sensors using Mobility Lab software (APDM Wearable Technologies, a Clario Company), and measures were extracted using custom algorithms written in Python (Wilmington, DE), available from the authors upon request.

After each in-person visit, participants wore the smartwatch on their more affected side and tracked symptoms on the smartphone daily for at least one week. Accelerometry data and tremor scores were collected from the smartwatch via Apple’s Movement Disorders Application Programming Interface (developer.apple.com/documentation/coremotion/getting_movement_disorder_symptom_data). Tremor analysis was performed on participants with at least 24 h of passive data over two weeks after baseline. The Movement Disorders API (open source code available at https://github.com/ResearchKit/mPower) generates tremor classification scores (none, slight, mild, moderate, strong, or unknown) for each 1-minute period, and the fraction of time spent in each category was calculated for each participant.

Using the BrainBaseline™ App, a cognitive and psychomotor battery was administered via the smartphone that included the Trail Making Test, modified Symbol Digit Modalities Test, Visuospatial Working Memory Task, and two timed fine motor tests (Supplemental Fig. 1)^40–42^.

Speech tasks included phonation, reading and a diadochokinetic task (not analyzed here)^43^. Phonation and reading files were processed using custom Python code (available from the authors upon request) with features computed using the Parselmouth interface to Praat and the Librosa library^44,45^. Common speech endpoints, such as jitter, shimmer, pitch statistics and Mel Frequency Cepstral Coefficients (MFCC), were computed. Speech segmentation was performed and used to extract time-related features for reading tasks^46^.

Participants also completed traditional rating scales including the MDS-UPDRS Parts I-III, Montreal Cognitive Assessment 8.1, Modified Hoehn and Yahr, Geriatric Depression Scale, REM Sleep Behavior Disorder Questionnaire, Epworth Sleepiness Scale, Scale for Outcomes in Parkinson’s Disease for Autonomic Symptoms, and the Parkinson’s Disease Questionnaire-8^47–54^.

### Study size

The study was powered to detect a mean change over 12 months for a digital endpoint with superior responsiveness to MDS-UPDRS Part III. The mean change in part III from baseline to year one in individuals with early, untreated PD in the PPMI study was 6.9 with a standard deviation of 7.0^3^. Allowing for up to half of the participants to begin dopaminergic therapy over 12 months and 15% drop out, the study aimed to recruit at least 75 participants with PD to yield 30 participants completing the study off medication. The study had more than 95% power to detect a true change of 6.9 units using a one-sample t-test and a two-tailed 5% significance.

### Statistical methods

For each gait feature, PD participants were compared to controls, and to gait scores from the MDS-UPDRS, using the Wilcoxon rank-sum test. The relationships between the gait features derived from smartwatch, smartphone and comparable features from the APDM sensors were estimated using linear regression. The relationship between the passive tremor fraction and tremor scores from the MDS-UPDRS was estimated using linear regression. Pearson correlations were determined to assess relationships between the digital cognitive battery and traditional cognitive measures. For each speech feature, normality was assessed using the Shapiro-Wilk test and transformed if necessary^55^. Features within each speech task with greater than 90% correlation were eliminated and remaining features were analyzed, with sensitivity analysis to understand sex differences. The AUC metric was used to determine how well each feature separated PD and control participants, and was computed for all participants by sex^24,56^.

Statistical analyses were performed using SAS v9.4, R v4.1, and Python 3.8. *P*-values < 0.05 were considered statistically significant. Results are considered exploratory and no adjustment for multiple comparisons were made.

### Missing data

If a participant had missing data for an outcome or as part of a necessary algorithm, that person was excluded for that analysis. Values of zero (i.e., did not attempt the task) were also excluded. Detailed reasons for missing data are outlined in Supplemental Table 3.

### Reporting summary

Further information on research design is available in the Nature Research Reporting Summary linked to this article.

## Supplementary information

Supplemental Tables and Figures

Reporting Summary

## Data Availability

The data are available to members of the Critical Path for Parkinson’s Consortium 3DT Initiative Stage 2. For those who are not a part of 3DT Stage 2, a proposal may be made to the WATCH-PD Steering Committee (via the corresponding author) for de-identified baseline datasets.

## Code availability

Custom Python code used for feature extraction is available from the authors upon request.
