# Supplementary material for: Using a smartwatch and smartphone to assess early Parkinson’s disease in the WATCH-PD study
Source: NPJ Parkinsons Dis. 2023 Apr 17;9:64. doi: 10.1038/s41531-023-00497-x (PMC10108794; doi:10.1038/s41531-023-00497-x)
Supplement: Supplementary file 1 — Supplemental Tables and Figures [file 41531_2023_497_MOESM1_ESM.pdf]

Supplemental Tables and Figures

Supplemental Figure 1. Flow of research participants
Supplemental Table 1. Comparison of cognitive and speech performance on smartphone assessments between those with and without Parkinson's disease
Supplemental Table 2. Description and location of assessments conducted with the digital devices used in the study
Supplemental Table 3. Sources of missing data

Supplemental Figure 1. Flow of research participants

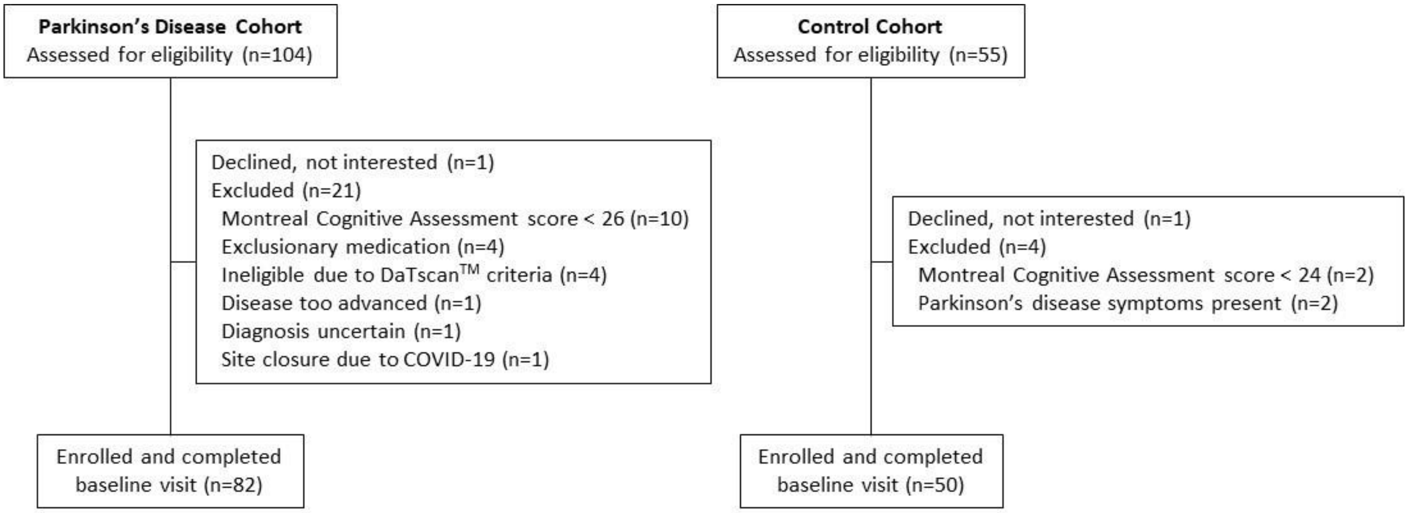

Supplemental Table 1. Comparison of cognitive and speech performance on smartphone assessments between those with and without Parkinson's disease

	PD cohort	Control cohort	p-value
**Cognitive Assessments**			
Trails Making Test Part A, s (PD = 82, controls = 49)	54.4 (23.8)	48.0 (36.0)	<0.01
Trails Making Test Part B, s (PD = 82, controls = 49)	55.6 (24.0)	48.6 (23.7)	0.11
Symbol Digital Modalities Test, number correct (PD = 82, controls = 49)	18.3 (8.2)	20.3 (8.9)	0.05
Visuospatial Working Memory Test, percent correct (PD = 82, controls = 49)	71.2 (21.6)	68.9 (26.7)	0.7
**Speech Assessments**			
Reading task, semitone pitch range (PD = 73, controls = 44)	4.6 (1.2)	5.6 (1.2)	<0.001
Reading task, cepstral peak prominence measure of vocal clarity (PD = 73, controls = 44)	27.4 (0.8)	27.8 (0.7)	0.003
Reading task, pause median length, log-transformed (PD = 73, controls = 44)	-1.5 (0.5)	-1.8 (0.6)	0.02
Phonation task, Mel Frequency Cepstral Coefficient 2 (PD = 51, controls = 40)	108.4 (17)	117.3 (17)	0.013

Results are mean (standard deviation)
PD = Parkinson's disease

Supplemental Table 2. Description and location of assessments conducted with the digital devices used in the study

Device	Assessment	Description	Location
Wearable Sensors[a]	Timed Walk Test	The participant is timed while walking for a distance of 10 meters. The individual walks the 10-meter path back and forth, turning at the end of their path, for 2 minutes.	In-clinic
	Repeat Timed Walk Test with Serial Sevens	The participant repeats the Timed Walk Test described above. While walking, the participant performs a serial subtraction of sevens beginning with the number 100.	
	Sit-to-Stand Test	The participant sits against the back of a chair and stands up as quickly as they can for 5 repetitions without stopping.	
	Postural Sway	The participant stands still, looking straight ahead for 30 seconds.	
Smartphone Application	Symptom Tracker	The participant answers a 5-item survey on the phone including questions about mood, sleepiness, thinking, tremor severity, and difficulty with movement.	In-clinic and remotely biweekly
	Symbol Digit Modalities Test	The participant is given a key that connects symbols to numbers. The participant is presented with a symbol and must speak aloud the corresponding number.	
	Trail Making Test	The participant must connect a set of dots as quickly as possible using the index finger on their dominant hand while still maintaining accuracy.	
	Visuospatial Working Memory	The participant is briefly shown four colored boxes. The participant is then shown a single-colored box and must indicate if that box was in the previous set of four.	
	Finger Tapping	The participant performs rapid alternating finger movements by tapping two targets that appear side by side using their index and middle fingers.	
	Fine Motor Test	The participant is presented with a pink object and an outline. The individual must use 1-2 fingers to move and rotate the object into the outline as quickly as possible.	
	Speech Assessment	Participants must perform a sustained phonation task, a verbal articulation task repeating the syllables “pa ta ka,” and a sentence reading task.	
Smartwatch	Timed Walk Test	The participant must walk in a straight line, turning at the end of their path, for 1 minute.	In-clinic and remotely biweekly
	Balance Test	The participant must stand still with their arms at their side for 30 seconds.	
	Tremor Task	The participant must rest their hands in their lap for 10 seconds, then extend their arms out in front of them for 10 seconds.	

[a]APDM sensors are also worn during the MDS-UPDRS part III and smartphone application tasks

Supplemental Table 3. Sources of missing data

Domain	Task	Explanation
Gait	Walking	About 25% of the missing smartphone data and 20% of the missing smartwatch data were because of unavailable files for analyses, whereas the remaining were the result of the gait analysis algorithms unable to generate metrics.
Tremor	Tremor	Missing passive tremor data were lost due to incorrectly configured permissions settings on the study phone.
Speech	Phonation	iPhone settings during early data collections (mostly in PD participants) included a form of lossy compression (Speex), which treated sustained phonation as background noise and cancelled out the signal of interest, greatly impacting acoustic metrics. This issue did not affect other speech tasks (pataka, reading). This issue was resolved early in the trial by a software update that switched to lossless compression.
	Reading	Audio recordings are missing for several subjects, likely due to early time out of the app session.
Psychomotor	Finger-Tapping	Missing data occurred due to issues with setting up the smartphone app at the Baseline visit that allowed the session to time out early.
	Fine Motor	
Cognition	Trails A & B	
	Symbol Digit Modalities Test (SMDT)	
	Visuospatial Working Memory (VSWM)	
